# Supplementary material for: Adverse events following COVID-19 vaccination: A comprehensive analysis of spontaneous reporting data in Ghana
Source: PLOS Glob Public Health. 2024 Sep 27;4(9):e0003770. doi: 10.1371/journal.pgph.0003770 (PMC11432875; doi:10.1371/journal.pgph.0003770)
Supplement: Attachment — Submitted filename: Response to Reviewers.docx [file pgph.0003770.s001.docx]

**RESPONSE TO REVIEWERS - [PGPH-D-24-01195] - [EMID:b7c1a6cfd1520acb]**

PGPH-D-24-01195
Adverse events following COVID-19 vaccination: A comprehensive analysis of spontaneous reporting data in Ghana


**JOURNAL REQUIREMENTS:**

**Comment**

1. Please send a completed 'Competing Interests' statement, including any COIs declared by your co-authors. If you have no competing interests to declare, please state "The authors have declared that no competing interests exist".

**Response**

*The authors have declared that no competing interests exist.*

**Comment**

2. Please amend your detailed Financial Disclosure statement. This is published with the article. It must therefore be completed in full sentences and contain the exact wording you wish to be published.

a. Please clarify all sources of funding (financial or material support) for your study. List the grants (with grant number) or organizations (with url) that supported your study, including funding received from your institution.

**Response**

No specific funding was received for the study. The Ghana FDA provided their support by providing the spontaneous COVID-19 AEFI data used for the analysis.

**Comment**

b. State the initials, alongside each funding source, of each author to receive each grant.

c. State what role the funders took in the study. If the funders had no role in your study, please state: “The funders had no role in study design, data collection and analysis, decision to publish, or preparation of the manuscript.”

If you did not receive any funding for this study, please simply state: “The authors received no specific funding for this work.”

**Response**

The authors received no specific funding for this work.

**Comment**
3. Please review your reference list to ensure that it is complete and correct. If you have cited papers that have been retracted, please include the rationale for doing so in the manuscript text, or remove these references and replace them with relevant current references. Any changes to the reference list should be mentioned in the rebuttal letter that accompanies your revised manuscript. If you need to cite a retracted article, indicate the article’s retracted status in the References list and also include a citation and full reference for the retraction notice.

**Response**

The Reference list has been updated with references that were used in the discussion section on vaccine-related sAEFIs. Refer to the Reference list numbers 25, 26, 28 (on page 29) and 53 (on page 32).

Also, some references have been corrected: refer to the Reference list numbers 27 (on page 29) and 33, 34, 35 (on page 30),

**REVIEWERS' COMMENTS:**

Reviewer's Responses to Questions

**Comments to the Author**

1. Does this manuscript meet PLOS Global Public Health’s publication criteria? Is the manuscript technically sound, and do the data support the conclusions? The manuscript must describe methodologically and ethically rigorous research with conclusions that are appropriately drawn based on the data presented.

Reviewer #1: Yes

Reviewer #2: Yes

Reviewer #3: Yes

**Response**

*Thank you.*

2. Has the statistical analysis been performed appropriately and rigorously?

Reviewer #1: Yes

Reviewer #2: Yes

Reviewer #3: I don't know

**Response**

*Thank you.*

3. Have the authors made all data underlying the findings in their manuscript fully available (please refer to the Data Availability Statement at the start of the manuscript PDF file)?

The PLOS Data Policy requires authors to make all data underlying the findings described in their manuscript fully available without restriction, with rare exception. The data should be provided as part of the manuscript or its supporting information or deposited to a public repository. For example, in addition to summary statistics, the data points behind means, medians and variance measures should be available. If there are restrictions on publicly sharing data—e.g. participant privacy or use of data from a third party—those must be specified.

Reviewer #1: Yes

Reviewer #2: Yes

Reviewer #3: Yes

**Response**

*Thank you.*

4. Is the manuscript presented in an intelligible fashion and written in standard English?

PLOS Global Public Health does not copyedit accepted manuscripts, so the language in submitted articles must be clear, correct, and unambiguous. Any typographical or grammatical errors should be corrected at revision, so please note any specific errors here.

Reviewer #1: Yes

Reviewer #2: Yes

Reviewer #3: Yes

**Response**

*Thank you.*

**RESPONSE TO COMMENTS FROM REVIEWER 1**
Please use the space provided to explain your answers to the questions above. You may also include additional comments for the author, including concerns about dual publication, research ethics, or publication ethics. (Please upload your review as an attachment if it exceeds 20,000 characters)

Reviewer #1: Thank you for the opportunity given me to review this paper which was well written.

**Comment**
Introduction
- Line 56: The authors may consider revising “method of injection” to “method of administration”. Since one is still in the introduction section and speaking in general terms, it may be wise to not limit yourself to a single mode of vaccine administration as different modes of administration will have different impacts on certain individuals.

- Authors refer to the deployment of a mass COVID-19 vaccination programme. Consider adding the date the mass vaccination programme kicked off in Ghana.

- You may also want to consider adding some context around when each of the various vaccines were introduced during this mass vaccination programme or were they all granted emergency use authorization at the same time in the country?

**Response**

- Line 58 (page 3) has been revised to read ‘*method of administration.*’
- The commencement date of the mass vaccination campaign in Ghana has been indicated *as 2^nd^ March 2021.* Please refer to line 70, page 4.
- The date of approval for Emergency Use Authorization (EUA) of the vaccines has been indicated as *20^th^ December 2021* (refer to lines 89-90, that is page 4).

**Comment**

Study Design
- It may be useful to explain the rationale for choosing the cut-off dates of 2nd March 2021 to 30th June 2022 for the data used in this study. For example, was the 2nd of March the date the 1st spontaneous report was received? Or was this period the period of the mass vaccination programme.

**Response**

Thank you for the suggestion. The underlisted statement has been included under the Study design, page 6, lines 135 - 138:

*‘The date of 2^nd^ March 2021 was when the mass vaccination campaign began in Ghana and 30^th^ June 2022 was chosen as the cut-off date since approval to the request for use of the FDA’s safety data was given in June 2022’.*

**Comment**

Study variables
- Data collection and handling processing
o Line 166: Please add the version of MedDRA that was used.

**Response**

The version of MedDRA used was version 24.0. Please refer to page 8, line 175.

**Comments**
Results
- Line 199: The authors may want to rephrase this sentence to make it clearer; firstly, when you talk about most reports, which reports specifically are you referring to (the total of 8498 or only the spontaneous reports)? You also outline most reports coming from the 5 different vaccines and it’s a bit difficult to understand the 83.6%. And lastly, if most reports came from the aforementioned 5 vaccines, where did the other reports come from?
- Table 1:

**Response**

Thank you for the comment. Lines 205 – 208 (page 9) have been rephrased to clarify that reference was being made to the 4,295 spontaneous reports which were found in the COVID-19 vaccine safety database. The other AEFIs which were reported through active surveillance were excluded from the data analysis as this was not the focus of the study.

*‘A total of 8,498 AEFI reports were obtained from the FDA’s COVID-19 safety database, out of which 4,295 were identified as having been spontaneously reported by vaccine recipients and healthcare professionals. The other AEFIs had been reported through active surveillance but were excluded from the analyses as this was not the study’s focus’.*

**Comments**

- The authors may want to consider adding a footnote explaining the 0% of dosage 2 for the J&J vaccine to remind readers that this vaccine requires only 1 dose for a primary vaccination series.

- It may be easier to read if the totals of the medical conditions are arranged in order of most common to least common with other medical conditions last. A footnote describing what constituted “other medical conditions” will also be helpful.
- Table 2: The authors may want to consider re-arranging the total types of AEFIs in order of most to least common with Other AEFIs last.

**Response**

- A footnote has been included for Table 1 indicating that the *0%* *recorded for the 2^nd^ dose of the J&J COVID-19 vaccine is because the vaccine is given as a single dose in the primary vaccination series.* Please refer to lines 220 – 221, page 13.
- For Table 1, the medical conditions have been re-arranged in the order of most common to the least common and ‘Other Medical Conditions’ last on the list (refer to pages 11 – 12). Also, a footnote describing ‘Other Medical Conditions’ has been inserted as follows (refer to lines 222 - 223, page 13):

*‘Other medical conditions include Eye problems, rashes, rheumatism, prostate enlargement, anaemia, diarrhoea etc.’*

- For Table 2, the AEFIs have been re-arranged in the order of most common to the least common and ‘Other AEFIs’ appearing last on the list. Please refer to line 229, page 14. – 15.

**Comments**
Serious AEFIs
- A major thing missing from this section is a detailed description of the diagnoses of the serious AEFIs reported, especially the 8 that were deemed to be causally related to the vaccines. One suggestion would be to describe them by vaccine type, dose, seriousness criteria (deaths, hospitalizations, etc) and outcome. The justification for this is that even though most AEFIs are mild, it’s the very few serious ones that contribute more to vaccine hesitancy and require more targeted public health messaging. So having more information on these will be important.

**Response**

Thank you for the suggestion. A short narrative has been included under the results section as indicated below to provide more details on the 8 vaccine-related sAEFIs (please refer to page 20 lines 296 – 302.

***Vaccine Product-Related Serious AEFIs***

*Eight (8) serious AEFIs were assessed by the FDA’s Joint COVID-19 Vaccine Safety Review Committee to be causally related to the vaccines. Of these, 5 were diagnosed as febrile illness related to the AstraZeneca vaccine and 1 incident of cerebrovascular accident also associated with AstraZeneca. For the Moderna vaccine, there was 1 case of gastroenteritis and 1 case of Stevens-Johnson syndrome. Seven of these events occurred after the first dose except for Stevens-Johnson syndrome for which the dose number was not stated.*

**Comment**
Discussion
- Line 298-301: The authors attribute the low incidence of AEFIs amongst people that received the Pfizer vaccine to the low deployment of this vaccine in Ghana. However, from table 3, Pfizer was the 2nd highest vaccine administered. The data presented and the conclusion drawn from it do not seem to correspond.

**Response**

Thank you for the comment. The statement, *‘This low incidence of AEFIs in the Pfizer vaccine could be attributed to the low deployment of the vaccine in Ghana’* has been deleted from the article (refer to lines 323 – 325).

**Comment**
- The discussion section is silent on how the serious AEFIs (especially those considered vaccine-related) compared with other studies.

**Response**

Thank you for the comment. A section has been added to the discussion providing more details on the 8 serious AEFIs which were causally related to vaccines (refer to pages 24, lines 385 – 394).

*The eight serious AEFIs which resulted in hospitalization were assessed by the FDA’s Joint COVID-19 Vaccine Safety Review Committee using the WHO Guidelines on Causality Assessment of AEFIs as having a Consistent causal association to immunization (A1: Vaccine product-related) ^(34)^. Of the eight cases, five and one were diagnosed as febrile illness and cerebrovascular accident respectively with the AstraZeneca vaccine. The remaining two cases were gastroenteritis and Stevens-Johnson syndrome for the Moderna vaccine. Seven of these events occurred after the first dose except for Stevens-Johnson syndrome for which the dose number was not stated. The eight vaccine recipients who had serious AEFIs fully recovered. None of these serious AEFIs were considered new because they were reported in the summary of product characteristics for the two products ^(53)^.*

**Comment**
- In addition to the cross-sectional design, could the fact that only spontaneous reports from passive surveillance be also a reasonable limitation to this study? Active surveillance contributed to about half of all the reports received. Could these AEFIs differ significantly from the spontaneous ones as spontaneous AEFIs are notoriously underreported?

**Response**

Thank you for the comment. It is possible that the type of AEFIs from the active surveillance may be different from what was observed in the passive surveillance, and this is another study worth considering. An analysis of the active surveillance data would have to be carried out and results compared with that from the passive surveillance data to ascertain whether there is a difference between the AEFIs reported using both systems.

We have already pointed out this study design weakness in the Study limitation section of the discussion section as follows:

*‘This study is limited by its use of a cross-sectional design that relied on spontaneously reported outcomes/symptoms, a situation which may sometimes be subjective (information bias) and may not depict the real adverse events experienced’.* Refer page 24, lines 398 – 401.

**RESPONSES COMMENTS FROM	REVIEWER 2**

Reviewer #2: Thank you for the opportunity to review this manuscript titled “Adverse Events Following COVID-19 Vaccination: A Comprehensive Analysis of Spontaneous Reporting Data in Ghana.” This topic is a good area to research considering the short period in producing the COVID-19 vaccines, the lack of information about the clinical trials, and the misconceptions people had and continue to have about the vaccines.

**Comment**
Kindly consider the following review to strengthen your paper:
Abstract
Well-stated abstract. It gives a brief overview of the entire study.
Page 2, line 37: Please give the values for the results of the logistic regression for the statement “Factors associated with serious AEFIs were age (AOR. 95% CI. p-value) and vaccine type.”

**Response**

Thank you for the comment. Lines 37 – 39 (Abstract) have been updated to read, *‘Relative to those 50 years and above, the odds of serious AEFI were 60% less among those aged <30 years (aOR=0.40, CI: [0.19, 0.86], p=0.019).’*

This statement has also been included in the Discussion (refer page 24, lines 378 – 381).

**Comment**

Introduction
Well-discussed introduction from the perspective of what AEFIs is, how it was rapidly developed with few long-term follow-ups on the clinical trials, reported cases globally (prevalence), the commonly reported reactions in the context of the specific vaccines as well as the context-specific for Ghana, and the need for this study.

To further strengthen your introduction, please provide a brief description of how the reported adverse drug reactions were managed, either by local means, hospitalization, or self-limiting.

**Response**

Thank you for the suggestion. The AEFIs which required treatment were treated in line with the WHO guidelines on case definitions and treatments for AEFIs. However, most of the non-serious AEFIs were self-limiting and resolved on their own. The underlisted statement has been added to the introduction section. Please refer to pages 5, lines 101 – 103:

*The AEFIs which were reported and required treatment were treated in line with the WHO guidelines on case definitions and treatments for AEFIs ^(28)^. However, most of the non-serious AEFIs were self-limiting and resolved on their own.*

**Comments**
Materials and Methods
Page 6, line 132: Changed “employs” after “assessment” to “employed.”
Page 9, line 199: The number for AstraZeneca is omitted. Since the others were quoted, it is good you quote that too.
Page 13, line 227: Delete “the” before “those.”

**Response**

- The word *‘employs’ has been changed to ‘employed’ in line 140, page 7.*
- The number of AEFI reports received for AstraZeneca which was *3,590* has been indicated (please refer to line 209, page 10).
- The word ‘*the’* has been deleted (refer to line 243, page 16).

**Comments**

Discussion
Well-discussed results with supporting references.

**Response**

Thank you for the kind words.

**Comments**

Conclusion
It is very succinct and reflective of the purpose of the study.

**Response**

Thank you.

**RESPONSES COMMENTS FROM REVIEWER 3**

Reviewer #3: This is a good manuscript. I understand that the first vaccine safety trials were done elsewhere not in Ghana, kindly consider to capture data for the local context if available. So that you can have a true reflection of AEFIs based in Ghana.

**Response**

Thank you for the suggestion. Unfortunately, there is currently no publicly available local data on COVID-19 vaccine safety trials.
